# Current and emerging approaches for eliminating *Borrelia burgdorferi* and alleviating persistent Lyme disease symptoms

**DOI:** 10.3389/fmicb.2024.1459202

**Published:** 2024-09-13

**Authors:** Kashaf Zafar, Onyedikachi C. Azuama, Nikhat Parveen

**Affiliations:** Department of Microbiology, Biochemistry and Molecular Genetics, Rutgers New Jersey Medical School, Newark, NJ, United States

**Keywords:** Lyme disease, *Borrelia burgdorferi*, treatment, antimicrobials, post-treatment Lyme disease syndrome, PTLDS

## Abstract

Lyme disease is the most prevalent tick-borne infection caused by *Borrelia burgdorferi* bacteria in North America. Other Borrelia species are predominately the cause of this disease in Eurasia with some distinct and various overlapping manifestations. Consequently, caution must be exercised when comparing the disease and its manifestations and treatment regimens in North America and Europe. Diagnosis of the early Lyme disease remains difficult using the currently FDA approved serological tests in the absence of a reported tick bite or of erythema migrans in many individuals, non-specific initial symptoms, and the absence of detectable anti-Borrelia antibodies in the prepatent period of infection. Furthermore, it is difficult to distinguish persistence of infection and disease versus reinfection in the endemic regions of Lyme disease by serological assays. If early infection remains untreated, spirochetes can disseminate and could affect various organs in the body with a variety of disease manifestations including arthralgias and musculoskeletal pain, neurologic symptoms and anomalies, and acrodermatitis chronicum atrophicans (ACA) in Europe. Although most patients recover after antibiotic treatment, an estimated ∼10–20% patients in the United States show persistence of symptoms known as post-treatment Lyme disease syndrome (PTLDS). The causes and biomarkers of PTLDS are not well-defined; however, several contributing factors with inconsistent degree of supporting evidence have been suggested. These include antigenic debris, dysregulation of immunological response, bacterial persisters, or combination of these features. This review highlights currently employed treatment approaches describing different antimicrobials used, and vaccine candidates tried to prevent *B. burgdorferi* infection.

## Introduction

Lyme disease is the most common vector borne disease, especially in the United States and Europe. In the United states, it is caused by spirochetes, *Borrelia burgdorferi* sensu stricto with approximately half a million cases suggested to occur by the Centers for Disease Control and Prevention annually (Schwartz et al., 2021), whereas *B. burgdorferi* sensu lato group with species that include *B. garinii, B. bavariensis*, and *B. afzelii* are prevalent in Europe with the rate of Lyme disease variable in individual European countries (Rauer et al., 2020). The incidence of Lyme disease is on the rise due to a combination of factors, such as increased habitat range of the main vectors, *Ixodes scapularis* ticks in the USA and *Ixodes ricinus* in Europe and the intersection of human domiciles with ticks and animal hosts. In addition, extended warmer season and consequent tick activity caused by climate change permits the expanding population of ticks to sites previously considered too cold and has been now reported as the main driver for increased risk of tick-borne diseases (TBD) in humans (Adrion et al., 2015; Steere et al., 2016; Telford et al., 2024).

Broad spectrum antibiotics such as amoxicillin, ceftriaxone, cefuroxime axetil and doxycycline are often used for treatment of early to late Lyme disease at present. Longer treatment regimens sometimes results in considerable adverse effects in patients due to the disruption of host microbiome and the selection for antimicrobials resistance in off-target bacteria (Willing et al., 2011; Modi et al., 2014). Even then, concern remains among number of clinicians and researchers that some of these antibiotics do not fully eliminate *B. burgdorferi* (Embers et al., 2012; Radolf et al., 2012; Hodzic et al., 2014; Feng et al., 2015b). The most troubling fact is that ∼10–20% of the infected individuals treated with antibiotics show persistence of symptoms referred to as post-treatment Lyme disease syndrome (PTLDS), thus revealing the unmet need for the development of therapeutics that also diminish the persistent subjective symptoms. To prevent *B. burgdorferi* sensu lato infection, attempts are being made to use prominent surface antigen(s) as effective vaccines with several antigens currently in the clinical trials (ClinicalTrials.gov, 2024) to overcome these problems. For instance, the vaccine candidate VLA15 has shown great promise in the second clinical trial phase (Comstedt et al., 2017; Hajdusek and Perner, 2023). A review of currently used treatment approaches and potential prospectives for developing combination of existing or novel antimicrobials together with alternative treatment approaches to alleviate suffering of Lyme disease patients and improving their quality of life is presented here.

### Acute Lyme disease and possible preventative measures

Lyme disease presents a significant health concern globally, particularly in the endemic regions of Europe and North America where infected ticks and reservoir hosts are prevalent. As the leading tick-borne illness and the fastest-growing vector-borne disease in the United States, Lyme disease poses a substantial burden on public health systems and individuals in the endemic regions. The positive Lyme disease test results have been reported now from all 50 states of the USA (Lee-Lewandrowski et al., 2019). *B. burgdorferi*-carrying ticks are maintained in nature by white-footed mice, rabbits, squirrels etc. Despite increased awareness and efforts to control tick populations, the incidence of Lyme disease continues to increase, necessitating effective treatment strategies to mitigate its impact on humans and pets. Failure to promptly administer treatment usually results in disseminated infection, sometimes with serious complications (Wormser et al., 2006; Mead, 2015). Various studies have indicated that a prolonged duration between symptom appearance and treatment initiation correlates with long-term unfavorable outcomes in the Lyme disease patients (Shadick et al., 1999; Ljøstad and Mygland, 2010; Eikeland et al., 2013; Knudtzen et al., 2017).

Following exposure to an infected tick, Lyme disease symptoms appear within a few days to weeks. Some specific symptoms are associated with this stage of infection although all individuals may not exhibit the early signs (Rahn and Malawista, 1991). The primary symptom of acute Lyme disease is a skin rash known as erythema migrans. This rash commonly emerges at the position of tick bite and gradually expands over time, resembling a bull’s-eye pattern. Other symptoms at acute Lyme disease include non-specific flu-like symptoms, including chills, fever, fatigue, muscle and joint discomfort, headache, and swollen lymph nodes. Some individuals may also exhibit neurological symptoms like meningitis, facial paralysis, or nerve pain radiating from the spine. Acute Lyme disease can rarely results in heart-related symptoms such as palpitations, chest pain, or heart block (Centers for Disease Control and Prevention, 2019). Acute Lyme disease is diagnosed on a purely clinical basis if erythema migrans is present, otherwise, diagnosis is based on disease symptoms congruent with confirmatory serologic tests. United States Food and Drug Administration (FDA) has approved a two serological test regime that consist of either 2 independent enzyme linked immunoassays (EIA) or EIA and Western blot test usually run consecutively (Mead et al., 2019) to detect antibodies against *B. burgdorferi* (Dressler et al., 1993; Depietropaolo et al., 2005; Wormser et al., 2013).

The risk of contracting *B. burgdorferi* and other tick-borne pathogens can be reduced in most effective way by taking steps to prevent tick exposure (Committee on Infectious Diseases, 2000). Since *Ixodes* ticks are slow to transmit infection requiring ∼36 h of attachment, prevention measures include wearing protective clothing, using tick repellents, conducting daily tick checks, and quickly removing any ticks observed on the body (Eisen, 2021). The routine use of serological testing or antimicrobial prophylaxis immediately after a tick bite is not useful for preventing Lyme disease because antibodies against various pathogens including Lyme spirochetes detected by serological tests usually appear in 2–4 weeks after infection making these tests ineffective. In addition, testing at this stage unnecessarily increases the medical care costs. Prophylactic antibiotics use could result in the emergence of antibiotic-resistant bacteria; however, it might be appropriate to consider administering a single dose of doxycycline under certain conditions, such as when the local infection rate with *B. burgdorferi* is higher than 20% and the tick remained attached to skin for more than 36 h. In these cases, treatment is usually initiated within 72 h of tick removal. Prophylaxis after *I. pacificus* bite is generally considered unnecessary unless higher infection rates of infections exist in the region (Nadelman et al., 2001). Detection by the healthcare providers proficient in identifying ticks, especially engorged ones, is usually beneficial. Individuals reporting symptoms of tickborne diseases within 30 days need to seek medical attention for a timely treatment (Costello et al., 1989; Salzman et al., 1993; Wormser et al., 2006).

The effect of *B. burgdorferi* on the nervous system was reported earlier in approximately 40% of patients examined (Fallon and Nields, 1994). Neurological involvement often extends across multiple regions of the nervous system and manifestations may include headaches, sensitivity to light, abnormal sensations (dysesthesias), stiffness in the neck, and irritability and even meningitis, cranial neuritis, and radiculoneuritis, either independently or in conjunction (Fallon and Nields, 1994). In Europe, meningoradiculoneuritis that is also known as Garin-Bujadoux-Bannwarth syndrome, makes a frequent appearance during acute Lyme borreliosis in adults, typically occurring after erythema migrans signs (Hansen and Lebech, 1992; Kaiser, 1994; Oschmann et al., 1998). In children, the condition often manifests as isolated meningitis without radicular symptoms (Hansen and Lebech, 1992; Pfister and Wilske, 1994; Berglund et al., 1995; Christen, 1996; Koedel et al., 2015). Central nervous system involvement is not common and occurs in around 4% of Lyme neuroborreliosis cases (Hansen and Lebech, 1992; Oschmann et al., 1998).

Lyme carditis symptoms usually appear within a few days to up to a month (averaging 21 days) after the onset of the infection. This condition is mostly observed in the summer to fall months (Steere et al., 1980; Krause and Bockenstedt, 2013; Forrester et al., 2014). Recent studies suggest that Lyme carditis incidence may be lower than previously estimated 4–10% of the untreated patients (Forrester et al., 2014; Kwit et al., 2018). The highest incidence of Lyme carditis was reported in childhood and middle age, with young adult and middle-aged men remaining most affected. Although the reason for predominance among male population remains unclear, more severe exposure or higher susceptibility could be contributing factors (Kwit et al., 2018). Cardiac involvement in Lyme disease can manifest in various forms, most commonly as atrioventricular nodal block which can rapidly progress to complete heart block (Steere et al., 1980; Rubin et al., 1992; Welsh et al., 2012). Other manifestations include atrial and ventricular arrhythmias and could involve sinus node and distal conduction system (Van der Linde et al., 1990; Rey et al., 1991; Greenberg et al., 1997; Oktay et al., 2015). The infection by *B. burgdorferi* can result in pericarditis and acute myocarditis, which may lead to ventricular dysfunction (Petr Kuchynka et al., 2015). Although most individuals recover with supportive treatment and antibiotic therapy, rare cases with the fatal consequence of infection have been reported (Forrester et al., 2014).

### Late Lyme disease manifestations

**FIGURE 1:**
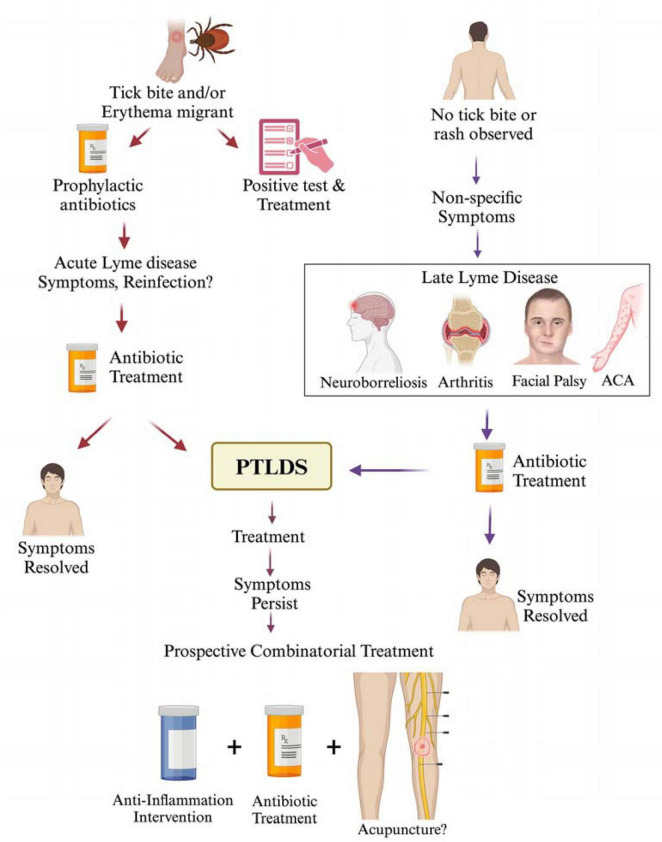
Lyme disease treatment at different stages of infection and PTLDS. A tick bite resulting in the development of erythema migrans is often a clear indication of Lyme disease in the endemic regions. In some cases, early infection can be treated prophylactically without a confirmed diagnosis of Lyme disease; however, antibiotic treatment is usually initiated after tests detect *B. burgdorferi* infection. Despite antibiotic therapy, some patients may continue to exhibit Lyme disease symptoms, which could be attributed to either reinfection or incomplete clearance of spirochetes. In such cases, additional antibiotic treatment is often administered with the commonly used antibiotics: doxycycline (100 mg, twice per day orally), amoxicillin (500 mg, three times per day orally) and cefuroxime (500 mg, twice per day orally). While Lyme disease symptoms resolve in most patients after completion of antibiotic treatment regimen, ∼10–20% report persistent symptoms or PTLDS, such as fatigue, cognitive impairment, joint and muscle aches, depression. In the absence of a documented tick bite history or erythema migrans but the presence of non-specific (subjective) symptoms, treatment is sometimes delayed, which could result in the development of late stage disseminated Lyme disease presenting a range of symptoms affecting the joints, nervous system, and skin. Treatment of late-stage Lyme disease often results in symptoms resolution or progression to PTLDS. Although no standard treatment is recommended for PTLDS at present, a combinatorial therapeutic intervention including antibiotics together with inclusion of either anti-inflammatory agents, or alternative treatments like acupuncture/electroacupuncture could be explored in the future to manage PTLDS.

Late Lyme disease refers to the advanced disseminated phase occurence when the infection remains untreated/inadequately treated during the early stage and typically appears in weeks, months, or even years after the initial tick bite. Late Lyme disease presents a range of symptoms that may affect various body systems, including the joints, nervous system, and heart. Managing the late stages of Lyme disease poses challenges due to diverse and sometimes debilitating symptoms, highlighting the importance of early detection and prompt treatment to prevent progression to this stage. We have summarized various disease manifestations and treatment approaches used at present for acute to late Lyme disease in Figure 1. According to the CDC, a case definition for confirmed late Lyme disease depends on signs depicting specific organ damage such as a clear neurologic disease or inflammatory arthritis with synovitis and joint effusion which are all confirmed by a positive western blot detection of IgG antibodies against *B. burgdorferi.* However, a significant proportion of untreated Lyme patients may only show symptoms like fatigue, myalgias or arthralgias without developing classical signs of neurologic impairment or Late Lyme arthritis (LA). It was previously believed that arthritis occurs in 60% of untreated cases of erythema migrans (Steere et al., 1987); however, surveillance data in recent years has shown that arthritis is presented in ∼30% of cases in the USA per year. It is important to note that lower percentage of patients likely experience LA because joint pain is sometimes mistaken as arthritis (joint inflammation).

LA patients do not always respond favorably to the first course of antibiotics treatment for 28 days with the presence of *B. burgdorferi* DNA in synovial fluid requiring additional 1–2 months of treatment (Marques, 2008). In patients with antibiotic-resistant LA, synovial fluid remained negative for *B. burgdorferi* DNA by PCR, but patients responded well to tumor necrosis factor (TNF) inhibitors or anti-inflammatory agents. Recent reports indicate that chemically atypical *B. burgdorferi* peptidoglycan (PG*^Bb^*) fragments shed into surrounding milieu during growth are a major contributor to inflammatory responses in LA, hence, potentiating other Lyme disease manifestations (Jutras et al., 2019). LA patients mount immunoglobulin G response against PG*^Bb^* which notably increases in the synovial fluid compared to that in the serum. This unusual peptidoglycan sugar arrangement in *B. burgdorferi* (noted by the presence of *N*-acetylhexosamine, Hex*N*Ac linked to *N*-acetylglucosamine in muropeptides) has also been reported to be resistant to lysozyme degradation and could be key to the extension of *B. burgdorferi* half-life in the synovial fluid of LA patients (DeHart et al., 2021). In view of these reports, the antimicrobials intervention specifically targeting this peptidoglycan could potentially be more effective in LA treatment.

Acrodermatitis chronica atrophicans (ACA) is a dermatologic appearance of late-stage Lyme disease associated with *B. afzelii* (Stanek et al., 2012). It is usually exhibited as a chronic, progressive skin disorder marked by skin atrophy, discoloration, and fibrosis that primarily affect the extremities, such as hands and feet. ACA is more commonly observed in the endemic regions of Lyme disease in Europe, particularly in the central and eastern countries. The condition is often associated with a long-term infection because it tends to develop months to years after initial exposure to Lyme spirochetes. Display of ACA could indicate the chronic nature of Lyme disease and need appropriate management and follow-up (Wormser et al., 2006).

## Treatment of Lyme disease

In the early stages of Lyme disease, adults with acute neurological symptoms such as meningitis or radiculopathy are advised to undergo intravenous (IV) treatment with ceftriaxone (Table 1). The recommended dosage of 2 g is administered once daily for a period of 14 days but could range from 10 to 28 days (Karlsson et al., 1994). In Europe, according to the methodological guideline of the Association of Scientific Medical Societies (AWMF), confirmation of Lyme neuroborreliosis (LNB) is through detection of inflammation in cerebrospinal fluid (CSF) attributed to the presence of Borrelia-specific intrathecal antibodies, thus suggesting blood-CSF barrier dysfunction. Garin-Bujadoux-syndrome (meningoradiculoneuritis) and acute facial nerve palsy with lymphocytic meningitis (without radicular symptoms) are common manifestations in adults and children, respectively and the recommended treatment in adults is with doxycycline or β-lactam antibiotics (cefotaxime, Penicillin G and ceftriaxone), which have proved to be effective in reducing neurological symptoms when treated for 14 days and 14–21 days respectively for early and Late Lyme neuroborreliosis (LLN) (Rauer et al., 2020). Penicillin G or cefotaxime could also be used as acceptable alternatives for parenteral therapy (Pfister et al., 1989). For patients intolerant to β-lactam antibiotics, oral doxycycline has been considered adequate. Children can be treated with ceftriaxone IV, or with cefotaxime or penicillin G at appropriate doses. Oral doxycycline has also shown success in this age group. Though antibiotic therapy may not accelerate seventh cranial nerve palsy resolution, it is essential to prevent further complications (Wormser et al., 2006).

**TABLE 1:** Recommended antimicrobials regimen for treating patients with Lyme disease according to CDC and AWMF guidelines.

Drug	Dosage for adults	Dosage for children	Duration (days)	References
**Recommendations in United States**				
Doxycycline	100 mg, twice/day orally (not suitable during pregnancy)	4.4 mg/kg/day orally, divided into 2 doses	10–14 (LD) 14–21 (Lyme Carditis, LLN) 28 (LA)	Nowakowski et al., 1995; Smith et al., 2002; Wormser et al., 2006; Kowalski et al., 2010; Stupica et al., 2012; Eliassen et al., 2018; Torbahn et al., 2018; Chason et al., 2019; Wormser et al., 2019a; Meissner and Steere, 2022
Amoxicillin	500 mg, 3 times/day orally	50 mg/kg/day orally, divided into 3 doses	14–21 (LD) 28 (LA)	Luger et al., 1995; Eppes and Childs, 2002; Smith et al., 2002; Wormser et al., 2006; Kowalski et al., 2010; Torbahn et al., 2018; Chason et al., 2019
Cefuroxime	500 mg, twice/day orally	30 mg/kg/day orally, divided into 2 doses	14–21 28 (LA)	Luger et al., 1995; Arnez et al., 1999; Eppes and Childs, 2002; Wormser et al., 2006; Cerar et al., 2010; Torbahn et al., 2018
Ceftriaxone	IV, 2 g daily	50–75 mg/kg daily	14–21 14–28 (LA)	Guet-Revillet et al., 2019; Dattwyler et al., 1987
**Recommendations in Europe**				
Penicillin G	IV, 20 million U daily	200–500 000 IU daily	14 (LD) 14–21 (LLN)	Mygland et al., 2010; Rauer et al., 2020
Doxycycline	200–300 mg daily orally (not recommended during pregnancy)	Age 9 above, 4 mg (max. 200 mg) daily.	14 (LD) 14–21(LLN)	Mygland et al., 2010; Rauer et al., 2020
Ceftriaxone	IV, 2 g daily	50 mg/kg daily	14 (LD) 14–21(LLN)	Mygland et al., 2010; Rauer et al., 2020
Cefotaxime	IV, 2 g × 3 g	100 mg daily	14 (LD) 14–21(LLN)	Mygland et al., 2010; Rauer et al., 2020
Amoxicillin	500 mg–1 g, orally	20 mg/kg/day orally	21 (LD)	Dattwyler and Luft, 1989; D’Alessandro et al., 2017
Cefuroxime axetil	500 mg, orally	15 mg/kg (max. 500 mg)	14–21 (LD)	D’Alessandro et al., 2017

Treatment may vary depending on patient’s, medical history, age, pregnancy status, underlying health conditions or allergies. For patients that are intolerant to amoxicillin, doxycycline, and cefuroxime can serve as alternatives. LD, Lyme disease (overall); LLN, Late Lyme neuroborreliosis; LA, Lyme arthritis.

Rebman and coworkers conducted a multivariant data analysis of the impact of treatment initiation time in early Lyme disease (Rebman et al., 2023). They divided the patients into two groups. The 1st group included the participants who displayed erythema migrans while the 2nd group of patients initially experienced flu like symptoms. Their analysis suggested that preliminary presentation of symptoms significantly affects “time-to-treatment” in early Lyme disease and found a significantly lower (22%) early treatment initiation time for erythema migrans participants than participants who had experienced various subjective symptoms. Overall, timely diagnosis and appropriate treatment in the early phases of infection generally resulted in a fast and complete recovery and prevented the progression to later stages of the Lyme disease (Cornell et al., 2020). The treatment regimens outlined in Table 1 are designed specifically for managing Lyme disease; however, modifications to these protocols might be needed depending on factors like medical history, age, underlying health conditions, susceptibility to various allergies or pregnancy (Smith et al., 2002; Wormser et al., 2006).

The efficacy of doxycycline for treatment was reported in a study involving 607 participants, with 93% of them receiving antibiotic in courses lasting 11, 11–15, or 16 days. Less than 1% of patients experienced treatment failure and more than half of the cases were due to reinfection (Kowalski et al., 2010). Doxycycline also has rare but possible side effects, including esophageal perforation, photosensitivity and pseudotumor cerebri (Angelette et al., 2023). A study comparing the effectiveness of doxycycline and cefuroxime in treating adult patients with erythema migrans found that 45/51 patients receiving doxycycline and 51/55 individuals taking cefuroxime exhibited satisfactory outcomes (Nadelman et al., 1992). A comparison of the efficacy of doxycycline and cefuroxime in treating children with erythema migrans showed that 92% of the children treated with cefuroxime experienced complete symptom resolution, compared to 67% of those treated with doxycycline (Eppes and Childs, 2002). Adults with skin rash in early Lyme disease are usually treated with a 14-day regimen of either amoxicillin at 500 mg three times per day, doxycycline with 100 mg twice per day, or cefuroxime axetil, 500 mg twice per day (Table 1). Treatment duration may vary for amoxicillin, cefuroxime axetil, and doxycycline with significant effectiveness in resolving early symptoms (Luger et al., 1995; Wormser et al., 2006; Eliassen et al., 2018). Macrolide are not typically recommended as the primary antibiotics for treating Lyme disease due to their reduced efficacy (Hercogová, 2023); however, they can still be considered for patients intolerant, or unable to take first-line antibiotics. Even then, patients need to be carefully monitored to assess the success of treatment employed for resolution of clinical symptoms (Labro, 1998; Boston, 2003; Hunfeld et al., 2004). First generation cephalosporins, such as cephalexin, lack efficacy in treating Lyme disease (Nowakowski et al., 2000).

Discussions among clinicians over the years about the ideal length of antibiotic treatment for Lyme disease (Wormser et al., 2003; Kowalski et al., 2010; Stricker and Johnson, 2010; Auwaerter et al., 2021) resulted in preference for shorter courses of antibiotics due to lower costs involved, decreased risk of side effects and because they minimize the chance of developing antibiotic resistance. For example, research on doxycycline effectiveness in treatment indicated that a 7-day course of antibiotics might be sufficient (Eldin and Hansmann, 2023). This finding is important for consideration by clinicians who may be reluctant to prescribe shorter antibiotic courses and have concerns that it may not fully clear the infection and cure patient.

The investigation of early Lyme neuroborreliosis was documented in eight randomized controlled trials (RCTs) and eight prospective cohort studies that examined the efficiency of antibiotic treatment duration (Oksi et al., 2007). For RCTs, antibiotics were generally prescribed for 14 to 21 days, with one trial extending the duration to 100 days (Oksi et al., 2007). In contrast, a prospective cohort study employed treatment durations ranging from 10 to 30 days, although time course of treatment was not specified in all studies. Regardless of the duration of their treatment, about 90% of patients experienced excellent or very good outcomes retained even more than 1 year later (Oksi et al., 2007). Selection of the antibiotic and understanding its associated outcomes is very important for an effective treatment strategy. A meta-analysis of data showed that oral doxycycline and the use of β-lactam antibiotics IV had comparable efficacy in improving neurological symptoms that remained effective after 12 months of infection (Dersch et al., 2015), supporting an earlier study (Halperin et al., 2007). A subsequent study of RCT investigated secondary endpoints, such as fatigue and quality of life, and found no significant differences between patients treated with doxycycline or β-lactam antibiotics after a 30-month period (Ljøstad et al., 2008; Eikeland et al., 2011). Two RCTs compared treatment of neurological Lyme disease and reported that cefotaxime caused a significant reduction in residual neurological symptoms between 4 and 12 months, while penicillin resulted in fewer side effects (Pfister et al., 1989; Hassler et al., 1990). The most common reported side effects of treatment were Herxheimer-like reactions and mild diarrhea; however, due to the limited reporting of major side effects, such as allergic reactions, shock and colitis, and the significant risk of bias in both studies, absolute recommendation for a single treatment approach could not be made (Dersch et al., 2015).

According to many studies, neurological symptoms were improved within weeks to months after receiving antibiotic treatment for 10–14 days. A prospective study involving 77 Bannwarth’s syndrome participants revealed that 88% of individuals reported positive outcomes until 12 months post-treatment (Ogrinc et al., 2016), consistent with the previous cohort studies that reported 90.6% symptom-free patients in 3 months after antibiotic therapy, and 95.2% with very good outcomes reported after a 33 months median follow-up (Hansen and Lebech, 1992; Kaiser, 2004). Another cohort study, which assessed predominantly early Lyme neuroborreliosis, found that 88% of patients’ daily activities remained unaffected after a 5-year observation period (Berglund et al., 2002).

There is a lack of high-quality trials specifically for evaluation of antibiotic treatment options for Lyme carditis, including choices of medication, methods of administration, and treatment duration. Therefore, current recommendations are based on varied studies with small patient groups for Lyme carditis, and on observational data (Dattwyler et al., 1997; Oksi et al., 2007; Pritt et al., 2016). A comparable efficacy assessment of oral doxycycline versus IV ceftriaxone in acute *B. burgdorferi* infection patients in a randomized controlled trial, where 6.5% of participants had carditis, showed that IV ceftriaxone was associated with a notably higher incidence of gastrointestinal side effects, while doxycycline was linked to a greater occurrence of dermatological adverse events (Dattwyler et al., 1997). Thus, Lyme carditis can be treated like the other manifestations of Lyme disease. Patients diagnosed with myopericarditis or atrioventricular heart block in the early stages of disease are usually cured with a 14-day course of oral or IV antibiotics. Hospitalization was suggested for symptomatic patients showing chest pain, dyspnea or syncope with continuous monitoring of patients with second or third level atrioventricular block, or those with prolonged PR intervals exceeding 30 ms needed, as the severity of blockage may rapidly worsen (Steere et al., 1980). For patients needing pacemaker, once the advanced heart block is resolved, recommendation included transition to oral antibiotics for completion of therapy and outpatient management (Pinto, 2002).

Early RCTs have shown that IV antibiotics are more effective in LA treatment in comparison to placebo (Steere et al., 1985; Caperton et al., 1990), with cephalosporins more effective than penicillin (Dattwyler et al., 1988; Hassler et al., 1990). Symptomatic patients’ treatment with non-steroidal anti-inflammatory drugs or corticosteroid injections was suggested if arthritis persisted despite antimicrobial therapy (Wormser et al., 2006). In studies by Steere’s group, 90% of children and adults patients’ arthritis resolved within 1–3 months when oral doxycycline (100 mg twice daily) or amoxicillin plus probenecid (500 mg every 6 h) was given (Steere et al., 1994; Steere, 2019). Although the results were not significantly different for Lyme neuroborreliosis, gastrointestinal unpleasant events and allergic reactions were higher in amoxicillin group. There are not any studies directly comparing the treatment with cefuroxime axetil with other oral antibiotics or placebo in treating LA. Oral antibiotics offer several advantages over IV antibiotics, including the ease of administration, fewer serious complications, and lower cost. Given their equivalent effectiveness, this approach using amoxicillin, doxycycline, or cefuroxime axetil antibiotic treatment of LA for 28 days is usually appropriate (Table 1). In late Lyme disease, adults showing neurological involvement of either the peripheral or central nervous system require careful management because some patients who received oral antibiotics exhibited clinical signs of neurological disease later (Steere et al., 1994; Steere, 2019) likely due to the dosing regimen and choice of antibiotic. Erythema migrans typically requires treatment for 2 weeks and acrodermatitis for a minimum of 4 weeks (Muellegger and Glatz, 2008). ACA is usually curable with a 21 day regime of commonly used antibiotics including doxycycline, amoxicillin, and cefuroxime axetil; however, a controlled study for treating ACA effectively has been recommended by clinicians to establish precise treatment regimen (Wormser et al., 2006). When treating spirochetal infections with antibiotics, patients may experience a transient Jarisch-Herxheimer Reaction (JHR), with typical symptoms including fever, nausea, vomiting, chills, tachycardia, headache, hyperventilation, hypotension, flushing, myalgia, and aggravation of skin lesions soon after initiation of therapy due to inflammatory immune response to antigens from dying organisms (Luger et al., 1995; Dhakal and Sbar, 2020). JHR is more common in relapsing fever *Borrelia* due to high bacterial burden in blood.

It is important to note that Lyme disease patients in endemic regions are equally at risk of coinfection with other pathogenic agents from *Ixodes* ticks such as *Babesia microti* and *Anaplasma phagocytophilum* which may prolong symptoms and complicate Lyme disease treatment (Hu and Shapiro, 2013; Parveen and Bhanot, 2019). In the United States, the reported rates of coinfection associated with either babesiosis or anaplasmosis in endemic regions ranges from 2–10 and 2–12% respectively and sometimes higher (Lantos et al., 2021). It is recommended that Lyme disease patients on antibiotic treatment, such as doxycycline with high grade fever for more than 1 day or abnormal laboratory characteristics like neutropenia, and/or anemia be investigated for possible coinfection with *B. microti and/or Anaplasma phagocytophilum*. Studies are in progress to track the expansion range of coinfecting pathogens and possible multiplex laboratory assays for simultaneously diagnosing multiple -infections (Hu and Shapiro, 2013; Chan et al., 2013). Comprehensive treatment approaches and regimens for Lyme disease during various coinfections are not yet streamlined.

### Treatment of pregnant women

The potential transmission of *Borrelia* spp. from mother to fetus has been an ongoing topic of discussion since the 1980s. Prior to the recognition of Lyme disease, cases of infantile illnesses showed clinical syndromes like Lyme disease. One infant, who initially displayed an erythema migrans rash, later developed symptoms including a painful and swollen right knee over a span of several months (Lampert et al., 1975). The Infectious Diseases Society of America issued guidelines depending upon evidence for Lyme disease (Wormser et al., 2000). The best antimicrobial therapy is still not fully established with doxycycline considered not suitable for pregnant women and during breastfeeding (Muanda et al., 2017a,b; Wormser et al., 2019b). Amoxicillin and third generation cephalosporins are considered safe and are preferred treatment options during pregnancy. The first-generation cephalosporins should be avoided because they have been found ineffective in both laboratory and clinical settings (Agger et al., 1992).

Timely treatment is essential for gestational borreliosis. Early localized infections are usually managed with a 14–21 day course of oral amoxicillin, taken at a dosage of 500 mg three times a day (Silver, 1997; Wormser et al., 2000). For patients who are allergic to amoxicillin, cefuroxime axetil at a dosage of 500 mg twice daily has been used as replacement. When neurological complications occur, IV ceftriaxone (2 g daily) or cefotaxime (2 g three times daily) orally is recommended for 14 to 28 days, the latter when only facial nerve palsy is observed with no signs of neuroborreliosis. Arthritis requires extended treatment with oral regimen lasting 30–60 days or IV treatment, depending on the severity of the condition. For first-degree atrioventricular block, oral treatment is often sufficient while more severe cardiac anomalies require IV therapy and cardiac monitoring. A review encompassing data from studies involving mothers who received antimicrobial treatment during pregnancy (Cook et al., 2023), and those who received no treatment (Markowitz et al., 1986; Lakos and Solymosi, 2010) found a significantly lower risk of adverse events when mothers received treatment during pregnancy with IV antimicrobial therapy yielding the lowest adverse outcomes. Conversely, the use of oral antimicrobials was associated with three times greater unfavorable outcomes, while the risk was six times higher in the untreated mothers. Additional studies without specified treatment protocols further supported a trend toward reduced (0.5–1.3%) adverse outcomes after antimicrobial treatment among large number of cohorts, while adverse outcomes among untreated mothers was found to be as high as 30 times greater (Williams et al., 1995; Strobino et al., 1999; Waddell et al., 2018).

Infections with *Borrelia* species during pregnancy can also be complicated by JHR, potentially impacting the fetus; however, JHR is less severe, uncommon, and generally has only a minor impact in Lyme disease (Butler, 2017; Nykytyuk et al., 2020; Trevisan et al., 2022). Karim and Sapadin (2023) reported a case of Lyme disease complicated by the JHR and coinfection with the parasite, *Babesia* in an endemic area (Karim and Sapadin, 2023).

## PTLDS causes and treatment approaches

Although antibiotics effectively cure most Lyme disease patients, ∼10–20% of patients report lingering symptoms, including depression, cognitive impairment, joint and muscle aches and fatigue (Melia and Auwaerter, 2016). These symptoms collectively known as PTLDS could persist for more than 6 months after completion of treatment regimen (Kullberg et al., 2020). Typically, alternative practitioners make this diagnosis, which has gained significant attention from the media and patient advocacy groups (Shapiro, 2014) while proof to support the notion that PTLDS results from persistent *B. burgdorferi* infection is lacking (Feder et al., 2007).

A systematic review of 687 patients with confirmed Lyme neuroborreliosis revealed the persistence of these post-treatment neurological symptoms in some patients (Dersch et al., 2016) with cranial nerve paresis (3.6%), sensory disorders (5.24%), extremity paresis (2.33%), pain (2.77%), and unsteady dizziness/gait/ataxia (2.62%). According to several studies, patients diagnosed with PTLDS often face difficulties in their daily lives, including work, social interactions, and family relationships (Zubcevik et al., 2020) and their overall quality of life resemble the individuals afflicted by other chronic conditions (Hill and Frost, 2022). Despite the significant impact of PTLDS, its causes, predisposing factors and biomarkers are not delineated, and therefore, effective diagnostic and treatment strategies remain challenging for the healthcare providers. PTLDS is only considered when Lyme disease infection was confirmed initially by a valid test conducted by reputable laboratories. Current evidence does not support that the persistent *B. burgdorferi* presence is associated with PTLDS. A history of Lyme disease results in re-administration of antibiotics in some PTLDS cases (Figure 1), prolonged antibiotic treatment is not recommended for patients experiencing ongoing subjective symptoms beyond 6 months (Klempner et al., 2013).

Post-antibiotic treatment LA, a well-researched aspect of Lyme disease, is believed to be due to autoimmunity and chronic inflammation (Steere, 2020). Several studies have investigated whether ongoing immune responses might contribute to prolonged symptoms following a Lyme disease diagnosis (Strle et al., 2014; Aucott et al., 2016). Elevated levels of immune modulators such as CCL19 and IL-23 have been observed initially in early stages of Lyme disease and were more common in patients with PTLDS (Strle et al., 2014; Aucott et al., 2016). Despite the initial presence of antibody responses, which declined over time, culture results were consistently negative, indicating no persistent active infection (Strle et al., 2014). Some patients with PTLDS showed mild elevations in C-reactive protein, an indicator of inflammation (Uhde et al., 2016) while others presented the elevated levels of serum antibodies reactive to neural antigens (Jacek et al., 2013). If PTLDS is caused by an autoimmune response, the improvement would only be possible with the use of immune-modulating therapies (Klempner et al., 2001). Central sensitization and the increased activity of nerve pathways reacting to sensory input after an infection, as reported after other infections (Hickie et al., 2006), could also play a role because many PTLDS symptoms resemble those of chronic fatigue syndrome and fibromyalgia. Therefore, treatments used for these infliction might also benefit PTLDS patients who meet the criteria for these overlapping conditions (Batheja et al., 2013).

A cohort study reported the lowest baseline QOL scores in the late-stage Lyme disease patients which later improved. The presence of underlying health conditions was significantly associated with lower QOL scores and long-term symptoms (Wills et al., 2016). A study conducted to assess Life Events Checklist (LEC) revealed that a higher exposure to previous traumatic life events was independently linked to an elevated risk of meeting PTLDS criteria; however, the study indicated that depression may not be the primary driver of increased symptoms reported (Aucott et al., 2022). In fact, surveys showed that pain, fatigue, depression, and quality of life in participants with the history of Lyme disease was significantly higher (13.7%) than the group of participants who did not have Lyme disease (4.1%), thus meeting the criteria for PTLDS. Furthermore, these individuals showed 5.28 times likelihood of developing PTLDS compared to those who did not have history of Lyme disease (Aucott et al., 2022). A population-based study revealed that one-third of Lyme disease patients faced delays in treatment, which was linked to the development of PTLDS (Hirsch et al., 2020). Avoiding delay in the medical care and initiating early treatment could overcome this problem.

There are no specific treatments currently recommended for PTLDS by The Infectious Diseases Society of America (IDSA), The American Academy of Neurology (AAN) and the Ad Hoc International Lyme Disease Group. Although there are many studies on extended use of antibiotic treatments for PTLDS, the specific antibiotics, duration, symptoms, and outcomes can vary across studies. Many studies identify cognitive deficits as a common symptom of PTLDS, frequently reported by patients themselves and probably due to the great impact on a person’s daily activities. Nevertheless, it’s important to consider patients’ perceptions of their illness because both subjective and objective cognitive deficits can profoundly affect a patient’s life and daily functioning. In a study in the northeastern United States on individuals who continued to experience symptoms after completing treatment for Lyme disease, participants were divided into two groups depending upon antibody positive test or erythema migrans lesion history. Both groups were split further into experimental and placebo groups, receiving either ceftriaxone and doxycycline or placebo. With initially, participants showed no significant differences in cognitive function, pain, role functioning, memory, or attention. After treatment, all groups improved equally no additional benefit from antibiotics, suggesting that improvements could be attributed to reduced pain and better mood, thus implying that self-reported symptoms may not always match objective testing and response to antibiotic treatment (Kaplan et al., 2003). Other placebo-controlled studies in patients with Lyme disease showed that a long term antibiotic use was ineffective for PTLDS (Klempner et al., 2001; Kaplan et al., 2003; Krupp et al., 2003; Fallon et al., 2008) and poses risks of severe side effects, including complications from IV catheters (Patel et al., 2000; Krupp et al., 2003; Wormser et al., 2006; Feder et al., 2007; Halperin et al., 2007; Berende et al., 2016; Goodlet and Fairman, 2018; Lantos et al., 2021). Reports of severe or fatal other bacterial infections after long-term antibiotic therapy further supports avoiding long-term antibiotics therapy (Marzec, 2017). Current research and recommendations of CDC on potential treatments for PTLDS indicated that patients treated with either oral or IV antibiotics faced higher risks of developing infections and electrolyte imbalances compared to those who did not receive antibiotic treatment (Goodlet and Fairman, 2018). A case study highlighted that ceftriaxone caused hemolytic anemia and acute kidney injury in a patient with PTLDS (De Wilde et al., 2017). These findings suggest antibiotics may not offer significant relief and recovery from PTLDS (Maksimyan et al., 2021). There is a need for new therapies to effectively address this problem including possibility of antimicrobial resistance emergence in *B. burgdorferi* contributing to development of PTLDS (Hodzic et al., 2008; Bockenstedt and Radolf, 2014).

The formation of persister cells of *B. burgdorferi* with increased tolerance to conventional antibiotics has also been reported (Sharma et al., 2015). In a time-dependent killing assay, a biphasic killing pattern showed the presence of persisters with majority of bacteria killed speedily, followed by a lower death rate of a subpopulation of persistent/tolerant spirochetes (Lewis, 2007; Maisonneuve and Gerdes, 2014). As with other investigated microbes, the fraction of *B. burgdorferi* persisters equally remains largely unchanged even with increased antibiotic levels. While some drugs, such as Daptomycin and Mitomycin C (though slightly toxic) were reported to eliminate both exponential culture as well as persister *B. burgdorferi* (Feng et al., 2014; Sharma et al., 2015), it is important to note that the development of persisters has only been shown *in vitro* without any evidence provided *in vivo*. Moreover, research is still ongoing to decipher the relationship between persister emergence and PTLDS.

### Human genotypes and immune responses to Lyme disease

Dysregulation of the immune response with tilting the balance toward proinflammatory cytokines production also plays a critical role in persistence of symptoms or PTLDS (Shen et al., 2010; Cerar et al., 2013; Vudattu et al., 2013; Strle et al., 2017; Wong et al., 2021). For instance, in a small nucleotide polymorphism (SNP) microarray analysis of 48 LA patients showed that arthritis resolved in 22 patients following antibiotic treatment (responsive) while 26 patients exhibited persistent arthritis after antibiotic therapy for 2–3 months (refractory) and ∼1200 SNPs varied in frequency (B allele frequency > 0.3) between these groups. The analysis of principal component of 28 SNPs showed complete separation in these groups, implicating human genetic variation influence on Lyme disease severity and persistence. Furthermore, top 10 SNPs that varied in frequency amongst the patients in refractory and responsive LA groups were correlated with the levels of crucial inflammatory mediators (IFNγ and IL-17) and anti-inflammatory cytokine IL-10 in their serum. Together these results reflected the association of certain human genotype mutations with persistent Lyme arthritis, although the mechanisms that influenced mutation and the exact outcome of these SNPs were not determined. Innate immune responses are often stimulated by Toll-like receptors (TLRs). On the cells surface, TLR1 forms heterodimer with TLR2 to recognize lipoproteins and triacyl-peptides. Studies have shown that TLR1 polymorphism enhanced lipopeptide response important during *B. burgdorferi* infection, and resulted in increased Th1 inflammatory response (Hawn et al., 2007; Strle et al., 2012).

An interesting study has shown a relationship between antibiotic refractory LA, the human histocompatibility leukocyte antigen (HLA)-DR4 molecules and the T-cell recognition epitope of *B. burgdorferi* OspA _(163–175)_ protein. Thus, a single spirochete peptide binding associated with certain HLA-DR molecules may be an indicator or a marker for refractory Lyme arthritis and could contribute to the disease pathogenesis (Steere et al., 2006). Furthermore, the role of pleomorphic forms of *B. burgdorferi* in Lyme disease pathogenesis may also play a role because round bodies were differently processed in the differentiated macrophages, and consequently, the immune responses were distinct, suggesting that spirochetes and round bodies possess diverse antigenicity and protein profiles and are likely contributors to Lyme disease pathogenesis (Meriläinen et al., 2016).

A recent human genome wide study explored phenotypic and genetic risk factors to identify the most prominent genetic variations association with susceptibility to Lyme disease (Strausz et al., 2024). The SCGB1D2 is usually present primarily in the skin, sweat, and other secretions. Interestingly, two known variants and an unknown common missense mutation located in the gene encoding secretoglobin family 1D member 2 (SCGB1D2) proteins were reported to enhance Lyme disease susceptibility. The *in vivo* inhibition of *B. burgdorferi* growth was observed after recombinant SCGB1D2 treatment in murine infection model. Results of this study suggested that normal SCGB1D2 protein could be a host defense factor that protects against Lyme disease.

### Metabolic enzymes of *B. burgdorferi* as potential drug targets

Genomic studies identify essential genes, enzymes, and metabolic pathways necessary for survival of *B. burgdorferi* in host. They can potentially be promising targets for drug development. For instance, computational *in silico* genome scale modeling of *B. burgdorferi’s* metabolism (iBB151) was constructed and the map was used to envisage important enzymatic reactions whose inhibition affected bacterial growth (Gwynne et al., 2023). Among 208 enzymatic reactions described, 77 were predicted to be essential for growth targeting the mevalonate pathway, alanine racemase, cell-wall synthesis, aminoacyl-tRNA ligases, adenosylhomocysteine nucleosidase and glutamate racemase pathways. When the predicted essential reactions from iBB151 (*B. burgdorferi* constructed model) were compared with *E. coli* (iML1515) and *Staphylococcus aureus* (iYS854) reaction models, 28 possible narrow spectrum drug targets for five major pathways (Folates, Glycolysis, Nucleotides Lipids and Mevalonate) specifically in *B. burgdorferi* were predicted. Among repurposed small enzyme inhibitor molecules, four were experimentally assessed. Theophylline and premetrexed were uniquely critical for targeting pyridoxal kinase and serine hydroxymethyl transferase enzymes, and inhibiting *B. burgdorferi* growth *in vitro*.

Recently, Hygromycin A (also referred to as totomycin), which is composed of modified cinnamic acid flanked by a furanose sugar and aminocyclitol, was isolated from the soil actinomycete *Streptomyces hygroscopicus*. This compound was smuggled into *B. burgdorferi* by a nucleoside transporter by binding to the conserved region of the 23S ribosomal RNA, thus targeting the peptidyl transferase center of bacterial ribosome to affect protein synthesis (Leimer et al., 2021). Hygromycin A exhibited activity against *Treponema pallidum*, (the causative agent of Syphilis), various species of *B. burgdorferi*, and other environmental spirochetes like *Alkalispirochaeta americana.* The selectivity of hygromycin A against spirochetes and its poor activity against intestinal bacterial isolates was shown by the treatment of *B. burgdorferi* N40 infected C3H mice with this compound. At clinically relevant doses, milder changes in gut microbiome with increased proliferation of symbiotic *Lactococcus* and *Lactobacillus* species was observed compared to amoxicillin and ceftriaxone treatment which produced blooms of pathogenic *Enterococcus* and *Bacteroides*.

Inhibition of metal acquisition and homeostasis by preventing metal uptake and utilization is another novel target for antimicrobial development. Manganese for instance, is a crucial co-factor for several protein and enzyme functions including superoxide dismutase machinery that protects *B. burgdorferi* from intracellular superoxide. Through a combination of *in silico* protein structure prediction and molecular docking, and screening of FDA approved libraries for potential compounds, which could bind to the metal transporter A (BmtA) predicted structure with great affinity, led to the identification of desloratadine and Yhohimbine which exhibited Borrelicidal activity *in vitro*. Treatment of the spirochete with desloratadine led to significant loss of intracellular Mn specifically and severe destruction of the bacterial cell wall (Wagh et al., 2015).

A critical regulatory and rate limiting enzyme, 3-hydroxy-3-methylglutaryl-coenzyme A reductase (HMGR) is associated with the mevalonic pathway, and contributes to a vital component necessary for peptidoglycan and cell wall biogenesis (Van Laar et al., 2012). Commercially available statins, such as simvastatin and lovastatin affected *in vitro* growth of *B. burgdorferi* MSK5 strain by inhibiting HMGR (Stupica et al., 2021) and reduced the burden of spirochetes in the mouse models. Moreover, treating infected mice with lovastatin notably upregulated several cytokines associated with the Th2 immune response, including IL-4, IL-5, IL-9, IL-10, and IL-13 (Van Laar et al., 2012; Van Laar et al., 2016) and likely decreased bacterial burden in the treated mice by either direct interference with spirochetal growth, modulation of the immune response, limitation of cholesterol availability to the spirochetes, or a combination of these mechanisms.

Methylthioadenosine (MTA)/S-adenosylhomocysteine (SAH) nucleosidases hydrolyses three substrates including 5-deoxyadenosine (5’dADO). The enzyme is present in many bacterial species but is absent in humans. MTA/SAH nucleosidases are also known to inhibit synthesis of polyamine and methyltransferase activities of many organisms (Parveen et al., 2006; Parveen and Cornell, 2011). *B. burgdorferi* is the only bacterial species identified till to date that possesses three homologues of MTA/SAH nucleosidase, Bgp, Pfs and MtnN, of which two are uniquely exported, functional proteins. These enzymes have been explored as effective drug targets in *B. burgdorferi* (Cornell et al., 2009; Chakraborti et al., 2020; Cornell et al., 2020). Among several substrate analogs and inhibitors tested *in vitro*, Formycin A and 5’-p-Nitrophenylthioadenosine (pNO_2_PhTA) showed Borrelicidal activities at rather low concentrations.

Since *B. burgdorferi* is auxotrophic for amino acids with few genomes encoding amino acid transport, peptides are important sources of amino acids essential for the regulation of virulence, cell division and morphogenesis. Groshong and colleagues structurally and transcriptionally evaluated the ability of *B. burgdorferi’s* oligopeptide transport system to import a variety of peptides during its enzootic cycle. The essentiality of peptides as major source of amino acids for *B. burgdorferi* was shown both *in vivo* and *in vitro* conditions. Authors concluded that peptides possess intracellular signaling functions that modulate cell division and morphogenesis. Depriving this transport affected the replication of spirochete indicating its potential as a target for drug development (Groshong et al., 2017).

### Vaccine candidates to prevent *B. burgdorferi* infection and Lyme disease

OspA is a lipoprotein on the outer surface of *B. burgdorferi* and is expressed during tick cycle. It can be targeted by antibodies to kill the organism in the tick midgut before transmission occurs. Research into vaccines for Lyme disease prevention has seen renewed interest in current years. LYMErix was the only licensed monovalent OspA serotype 1 based recombinant vaccine from *B. burgdorferi* sensu stricto in the United States from 1998 to 2002 which reduced Lyme disease infections in vaccinated adults by nearly 80%; however, the vaccine was discontinued due to concerns about the OspA triggering an autoimmune response that led to its low adoption rates. A multivalent OspA-based vaccine (VLA15), which targets various clinically relevant *Borrelia* species and OspA serotypes found in US and Europe, is composed of three proteins with each harboring the C-terminal fragment of OspA serotypes linked in pairs to form three fusion proteins. Triple immunization of mice with this vaccine protected them from infection when challenged with either *in vitro* grown spirochetes or those transmitted by infected ticks (Comstedt et al., 2014; Comstedt et al., 2015; Comstedt et al., 2017). Currently, Valneva SE and Pfizer Inc report positive Phase 2 clinical trial data for VLA15 with the phase three trials to start in a near future. VLA15 testing after two (0 and 6 months) or three (0, 2, and 6 months) administrations of primary series doses in 5–11, 12–17, and 18–65 year age groups showed higher success in the adult participants with age 18–65 years who received three doses compared to those who received two doses (Hajdusek and Perner, 2023).

Using the same approach as VLA15 vaccine, a prototype vaccine that makes the use of *Helicobacter* pylori ferritin nanoparticles fused with seven OspA serotypes from *B. garinii*, *B. bavariensis*, *B. afzelii* and *B. burgdorferi* exhibited long-lasting high-titer antibody response in both rhesus macaque and mouse infection models (Kamp et al., 2020). Other vaccine approaches explored include an OspA-encoding lipid nanoparticle-encapsulated nucleoside-modified mRNA (mRNA-LNP) vaccine that was recently assessed for protective efficacy and immunogenicity in comparison to alum-adjuvanted OspA protein subunit vaccine (Pine et al., 2023). In addition, a subunit vaccine containing a combination of OspA and 14 immunogenic linear epitopes (“chimeritope”) from different OspC isotypes (Marconi et al., 2020) has also been evaluated as a promising vaccine. In another experimental study, the outer surface protein, Cspz, associated with complement evasion was explored as a vaccine candidate (Marcinkiewicz et al., 2018). In an earlier study, a combined DbpA, BBK32, and OspC based vaccine was reported to show better protection against infection in mice compared to a single or double antigen vaccine (Brown et al., 2005).

Multiple tick-borne disease can also be prevented by developing anti-tick vaccines. Mice administered with antiserum to the tick salivary gland protein, Salp15, when subsequently challenged with *B. burgdorferi* were protected from tissue colonization (Dai et al., 2009). Salp15 was shown to protect *B. burgdorferi* against host immune responses through its binding to OspC (Ramamoorthi et al., 2005). It equally binds with CD4 co-receptor, inhibits CD4^+^ T-cell activation and alters the cytokines expression level (Anguita et al., 2002; Hovius et al., 2008). Interestingly, silencing of tick histamine release factor (tHRF), which is highly expressed in *I. scapularis* ticks infected with *B. burgdorferi* (Strnad et al., 2020), through RNA interference affected the efficiency of tick feeding with subsequent reduction in spirochetes burden in mice (Dai et al., 2010). Furthermore, a feeding-induced salivary protein known as tick salivary lectin pathway inhibitor (TSLPI) is found in *I. scapularis* (Schuijt et al., 2011) and *I. ricinus* (Wagemakers et al., 2016). The TSLPI-silenced ticks or ticks fed with TSLPI immunized mice blood were impaired in spirochete transmission. Additionally, the persistence and acquisition of *B. burgdorferi* in tick midguts was reduced when fed on TSLPI-immunized mice, implying an important role also in spirochete transmission to the mammalian host (Schuijt et al., 2011). Targeting tick proteins could elicit “tick immunity” or a situation whereby the host is resistant to tick bites and prevent infection by tick-borne pathogens. In fact, recent studies showed that immunization of animals with mRNA-lipid nanoparticle vaccine encoding 19 *I. scapularis* proteins offered acquired resistance to ticks (Sajid et al., 2021; Matias et al., 2023) offering a promising new approach to prevent transmission of not only Lyme spirochetes but also other tick-transmitted pathogens.

## Future prospective

Although some suggested causes remain controversial, persisting symptoms in patients following antibiotic treatment of Lyme disease could be associated with the presence of drug-tolerant persisters, antigenic debris, impaired immunological response or a combination of these culprits, but the root causes of the post treatment symptoms still remains unknown (Bobe et al., 2021). Some antibiotics act synergistically and could serve as a future therapeutic intervention in alleviating Lyme disease symptoms, such as trimethoprim and sulfonamide with aminoglycosides and β-lactams (Levin and Harris, 1975). One of the effective combinations against microcolony form of *B. burgdorferi* include daptomycin and doxycycline with a beta-lactam cefoperazone (Feng et al., 2015a)

The interaction of bacteria with tick proteins in the gut and salivary glands significantly influences the bacterial transmission. Targeting tick proteins or any step involved in tick transmission cycle could confer increased protection. Hence, anti-tick vaccine development could be seen as a promising approach to protect individuals against multiple tick-borne associated infections, including by *B. burgdorferi* (Bobe et al., 2021). Additionally, glycolipids such as acylated cholesteryl galactoside (ACGal) (Schröder et al., 2003; Stübs et al., 2009) and polymers consisting of sugars and peptides (Jutras et al., 2019) could serve as adjuvants or potential vaccine candidates.

Acupuncture has been used for centuries in Asian medicine to alleviate pain and inflammation. From a clinical perspective, ST36 (sciatic nerve) stimulation is the most common treatment in acupuncture for inflammatory and infectious disorders by acting through stimulation of vagal nerve (Ulloa et al., 2017). In a recent study, electroacupuncture could alleviate LA symptoms in mice although it didn’t affect spirochetes burden (Akoolo et al., 2022). Further reports highlight the effectiveness of acupuncture in alleviating Lyme disease symptoms like migraines and musculoskeletal pain. In a case of a 44-year-old female who underwent acupuncture alongside antibiotic treatment, significant symptom resolution was observed over 10 months suggesting that it could also reduce other Lyme disease persisting symptoms (Bartecchi, 2006; Adams et al., 2023). A novel therapeutic approach to treat PTLDS involved engineering Chimeric Antigen Receptor (CAR) T-cells to target and eliminate *B. burgdorferi*. The efficacy of these CAR T-cells needs to be tested *in vitro* and *in vivo* using reliable animal models before being administered to patients. This innovative proposal will need to establish a treatment protocol for PTLDS, which could offer relief and alleviate suffering of patients from this chronic and debilitating condition. A novel method of preventing tick-borne diseases using CRISPR-based genome editing technology is in pipeline (Buchthal et al., 2019) in which local populations of wild white-footed mice (chief reservoir host) will be immunized against Lyme disease and ticks using antibodies obtained from natural adaptive immunity. The overall goal of this project is to minimize host reservoir competence for a long time.

To summarize, treatment of Lyme disease could also be tailored to patients’ immune response system, genetic make-up, and other host-factors to minimize relapses, persistent inflammation or degeneration to chronic conditions which could improve outcomes. Interestingly, recent demonstration of specific fibroblast growth factor receptor inhibitors showed attenuation of neuroinflammation and apoptosis induced by non-viable and live *B. burgdorferi* from rhesus frontal cortex and dorsal root ganglion tissue explants (Parthasarathy, 2024). Overall, this study implies that inhibition of chemokines and cytokines could serve as therapeutic targets for patients with refractory neurological Lyme disease. In addition, public awareness in the form of education, surveillance and early intervention could go a long way to reduce burden of infection in the rural communities. There is also a need for technological remote monitoring of individuals in rural communities where Lyme disease is endemic. Although the rate of adverse outcome can be reduced by using antimicrobials, the best strategy is to prevent infection, especially during pregnancy. This can be accomplished by avoiding exposure to ticks and prompt removal to reduce the risk of transmission (Costello et al., 1989; Shapiro et al., 1992; Agre and Schwartz, 1993). The use of repellents like DEET is effective in tick bite prevention measure and has no known risk to the developing fetus (McGready et al., 2001; Koren et al., 2003). Some clinical practitioners prescribe amoxicillin for pregnant women, especially after documentation of prolonged tick attachment (>48 h) in highly endemic regions. Careful monitoring for symptoms of tick-borne illness could be required if prophylactic antibiotics aren’t given after tick exposure.

Finally, a combinatorial approach involving antibiotic and alternative treatment approaches like acupuncture or the use of anti-inflammatory agents in the cases of severe symptoms could be explored to alleviate suffering of patients due to post-treatment persisting symptoms and improving their quality of life.

## Conclusion

This review highlights the treatment approaches currently in use to eliminate *B. burgdorferi* infections and potential novel strategies to alleviate persistent Lyme disease symptoms. It is worth mentioning that the review of Lyme treatment and clinical studies can be cumbersome owing to the varying degrees of inclusion criteria in the definition of Lyme disease. PTLDS reviews are particularly limiting because a strict inclusion and exclusion criteria need to be identified. Some of the studies presented here are stringently restricted by Lyme infection diagnosis according to CDC approved two-tier serologic tests while others are slightly permissive. Even then, we have tried to include studies in a comprehensive manner to provide an overall perspective to researchers, clinicians and patients.
